# Conversion of failed revision total knee arthroplasty in arthrodesis with modular nail maintaining the uncemented femoral stem in patient with extensor mechanism insufficiency: a case report

**DOI:** 10.1186/s13256-024-04380-y

**Published:** 2024-03-09

**Authors:** Giulio Maria Marcheggiani Muccioli, Domenico Alesi, Vito Gaetano Rinaldi, Tosca Cerasoli, Davide Valente, Stefano Zaffagnini

**Affiliations:** 1 II Clinica Ortopedica-IRCCS Istituto Ortopedico Rizzoli, Via Pupilli, 1, 40137 Bologna, Italy; 2 Department of Biomedical and Neuromotor Sciences, University of Bologna, Bologna, Italy

**Keywords:** Knee arthrodesis, Revision total knee arthroplasty, Modular knee fusion nail, Periprosthetic knee fracture

## Abstract

**Background:**

The transition from revision total knee arthroplasty (RTKA) to arthrodesis involves the replacement of cemented femoral and tibial stems with a modular nail designed for arthrodesis. This conversion process is associated with challenges such as bone loss, blood loss, and prolonged surgical durations. Effectively addressing these complexities through a less invasive surgical approach could be pivotal in enhancing patient outcomes and minimizing associated complications.

**Case presentation:**

A 75-year-old white Caucasian female patient with a revision total knee arthroplasty (RTKA) performed with a modular uncemented rotating-hinge system, reporting an history of recurrent patellar dislocation, was referred to our institution after a fall resulting in periprosthetic tibial plateau fracture. The fracture was treated with open reduction and internal fixation, but afterwards the patient had been unable to walk again. Tibial stem was mobilized, and extensor mechanism was insufficient due to chronic incomplete quadriceps tendon rupture. The femoral stem was stable, so we decided to convert the rotating-hinge in a arthrodesis with an uncemented modular knee fusion nail maintaining the previous femoral stem.

**Conclusions:**

The result was a successful arthrodesis with minimal bone and blood loss, reduced operative time, and optimal functional outcome at the one-year follow-up. This case highlights the advantage of using a modular knee revision platform system that gives the opportunity to convert a RTKA in arthrodesis.

## Introduction

One of the most challenging complications associated with revision total knee arthroplasty (RTKA) is aseptic mobilization resulting from trauma. A periprosthetic fracture with a delayed consolidation and bone loss when combined with extensor mechanism insufficiency and muscle atrophy, leads to compromised limb function and severe pain, significantly impacting the patient's quality of life. In such cases knee arthrodesis emerges as the most suitable option, particularly for patients with functional hips and ankles [1, 2]. The conversion of an RTKA to arthrodesis involves the removal of the femoral and tibial stems and their replacement with a modular nail for arthrodesis. This usually leads to bone loss, blood loss, and long surgical times.

In this case, we describe a knee arthrodesis procedure using a modular tibial nail combined with the original femoral prosthetic stem. This approach was employed in a patient with aseptic RTKA tibial stem mobilization and extensor mechanism insufficiency following a periprosthetic tibial plateau fracture and an incomplete quadriceps tendon rupture.

## Case presentation

**Fig. 1:**
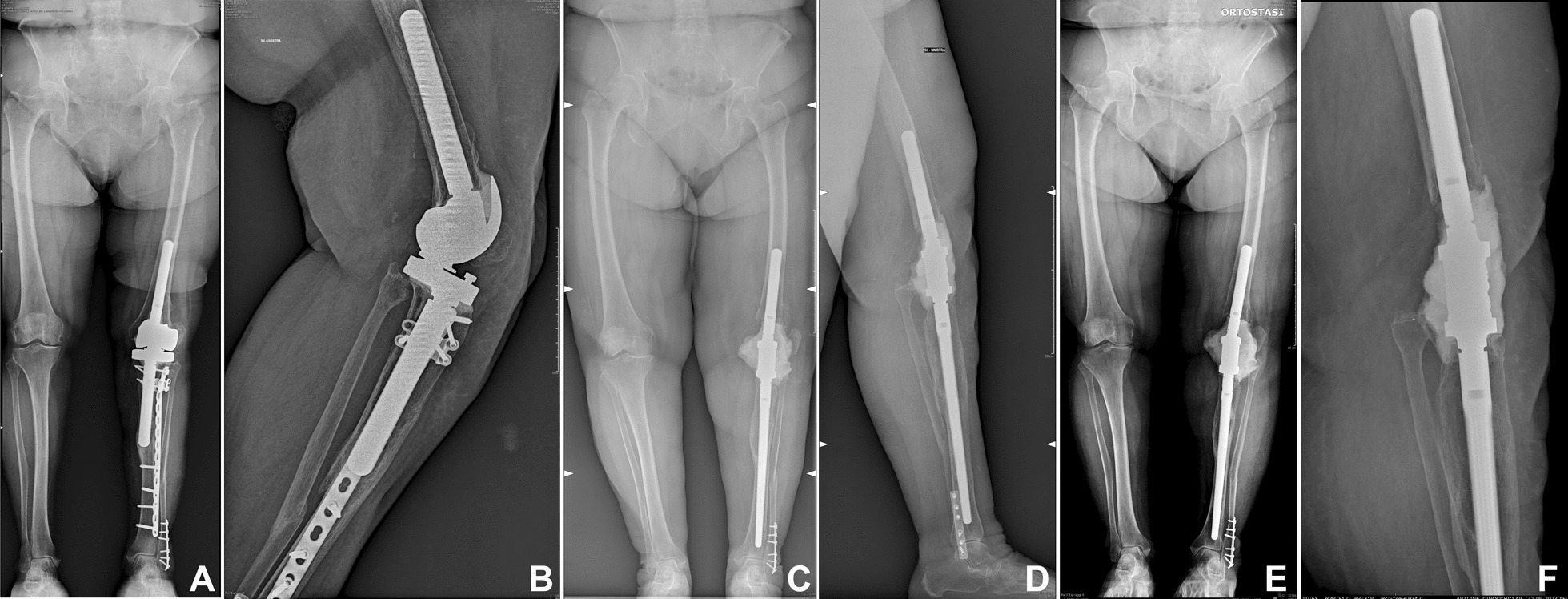
**A**, **B** Preoperatory X-rays, **C**, **D** X-ray at 21 days post-surgery, **E**, **F** 1-year follow up X-rays

A 75-year-old white Caucasian female patient presented to our clinic in August 2022, complaining of left knee pain and weight-bearing intolerance, even when using an extension knee brace and crutches. The knee was swollen with lateral patella dislocation and a passive range of motion of 0–120°. She underwent a medial unicompartmental knee arthroplasty (UKA) surgery in 2009 with good outcomes; then, in April 2021, she was diagnosed with left peroneal malleolus fracture, incomplete quadriceps tendon rupture with patellar dislocation, and instability of the UKA after a trauma. The fracture was treated with open reduction and internal fixation (ORIF) and the UKA was revised with a uncemented modular rotating hinge prosthesis (Endo-Model-M, LINK—Hamburg, Germany) in July 2021. Nevertheless, in December 2021, there was a recurrence of left patellar dislocation treated conservatively with a brace and weight-bearing restriction for one month. Then, in January 2022, after another fall, the patient experienced a Felix type III periprosthetic tibial fracture, treated with open reduction and internal fixation. Since this event, 8 months before being referred to us, the patient has been unable to walk. Radiographs performed at that time showed a tibial malunion with tibial stem mobilization due to stress-shielding (Fig. 1A, B).

To rule out infection, an arthrocentesis was performed, it showed clear yellow fluid without any traces of blood. The cell count values were 180 cells/microL, with 17% polymorphonuclear cells. Cultures were negative for both aerobes and anaerobes. The blood levels of C-reactive protein (PCR) were 2 mg/dL. At the time of the surgery the patient was affected by tibial malunion, aseptic loosening of the tibial component, extensor mechanism disruption with consequent patellar irreducible dislocation. The patient didn’t present any rotational defect,the patellar dislocation was entirely attributed to the rupture of the extensor mechanism with impaired healing and retraction of tissues that kept the patella laterally dislocated. The pre-surgery plan was to remove the tibial plate, screws, and all prosthetic components, replacing them with an uncemented modular knee fusion nail (Endo-Model Knee Fusion Nail SK, LINK—Hamburg, Germany). A lateral distal approach, centered on the previous scar, allowed for the removal of the distal tibial screws. An anterior medial approach to the knee, extended to the proximal part of the tibial diaphysis, was performed for a complete removal of the tibial plate and screws. Subsequently, an arthrotomy was performed. Five samples for culture examination were taken. The patella was irreducibly dislocated, necessitating a lateral release and the removal of its prosthetic component. The tibial prosthetic component was extracted through an anteromedial tibial window and, following appropriate preparation, a 240 mm non-cemented stem was inserted into the tibial canal. Despite numerous attempts, the femoral stem could not be mobilized due to its deep osteointegration, so only the femoral shield was removed. After confirming stability and positioning, arthrodesis was achieved by connecting the retained femoral stem with the modular interconnection of the knee fusion nail system. The proximal tibia was further stabilized with Tycron no.5 cerclages. After nail positioning, the 10 cm gap between distal femur and proximal tibia was filled with antibiotic cement spacer (Palacos-R, Heraeus Medical -Wehrheim, Germany) (Fig. 1C, D).

The limb was immobilized in a brace for the first 21 days post-surgery, after which the patient began exercises for hip and ankle mobilization, isometric muscle strengthening, and magnetotherapy. Non-weight-bearing ambulation was prescribed for 30 days. All intraoperative culture tests yielded negative results.

After three months, the patient was able to walk with just one crutch, and after six months, she was allowed to resume normal activities. At one-year follow-up, the patient can walk using a cane, enabling full weight-bearing on the operated limb. She reports an improved sense of well-being and a return to normal daily activities. The patient uses a 1.5 cm insole to enhance walking comfort and reduce pelvic tilting. follow-up X-rays confirmed the stability of fixation devices and cement, without signs of subsidence of the implant (Fig. 1E, F).

## Discussion

The conversion of a failed RTKA into an arthrodesis with a modular nail is a complex surgical procedure, in terms of achieved outcomes, operative time, blood loss, and bone loss. To the best of the authors’ knowledge, combining a femoral stem from a RTKA with a standard modular knee fusion nail has not yet been documented in the literature. The initial surgical plan aimed to remove all prosthetic components in order to allow the implantation of a new femoral stem of the same length of the tibial one to ensure a better force and stress distribution along the final fusion nail. Authors intended to use cementless press-fit stems as cement would prevent the tibial malunion from healing [1]. The tibial stem was very easy to remove due to the tibial malunion. Given the complex clinical presentation upon the patient's arrival, the authors made the decision not to pursue anti-resorptive therapy or physical therapy modalities, such as magnetotherapy, for the tibial malunion. This choice was made to prevent any additional delays in initiating definitive treatment. Differently from the tibial stem, the femoral stem showed a deep osteointegration, making its removal very difficult and harmful to the patient’s poor bone stock. Consequently, intraoperatively it was decided to perform an hybrid arthrodesis. The authors opted for arthrodesis over tibial component revision combined with extensor apparatus reconstruction using mesh due to the chronic complete rupture of the extensor apparatus, along with retraction of the quadriceps tendon, complicating and destabilizing the reconstruction. Additionally, the patient's clinical condition was exacerbated by eight months of non-weight bearing, contributing to sarcopenia, and coupled with cardiac comorbidities, lowering the functional demand. Moreover, a second RTKA surgery posed a higher risk of infection compared to conversion to arthrodesis. The author choice was guided also by the work of professor Froschen *et al*., who presented a case series of 4 patients with failed RTKA and extensor mechanism insufficiency treated with conversion in arthrodesis retaining the prosthetic components and using a custom-made modules [2]. The main difference was that no custom-made modules were needed in this case because the knee fusion nail (Endo-Model Knee Fusion Nail SK, LINK—Hamburg, Germany) provide a modular interconnection that perfectly fit whit the femoral stem of the prosthesis (Endo-Model-M, LINK—Hamburg, Germany).

It is widely recognized that while achieving a fusion rate of 95%, intramedullary nailing has its drawbacks, including extended surgical time and increased bone loss [3]. Furthermore, failed RTKAs often results from infection [4]. The risk of substantial bone loss, intra-operatory fractures, fatty emboli, and the spread of chronic infection, is significantly reduced when a stable stem from the previous non infected RTKA can be retained. However, it is crucial that this component is properly positioned and stable, and that arthrocentesis yields negative results for infection. Additionally, the use of antibiotic cement serves as a valuable prophylactic measure.

## Conclusion

This surgical case represents the first successful report of combining a non-cemented femoral stem from a RTKA with a modular knee fusion nail, resulting in a successful surgery with minimal bone loss and blood loss, shorter operative time, and optimal functional outcomes, at one-year follow-up. This case highlights the advantage of using a modular knee revision platform system who gives the opportunity to convert a RTKA in arthrodesis.

## Data Availability

All the data discussed in the manuscript are in the databases of Istituto Ortopedico Rizzoli.
